# A radiomics model for predicting the response to methylprednisolone in brain necrosis after radiotherapy for nasopharyngeal carcinoma

**DOI:** 10.1186/s13014-023-02235-2

**Published:** 2023-03-01

**Authors:** Xiaohuang Zhuo, Huiying Zhao, Meiwei Chen, Youqing Mu, Yi Li, Jinhua Cai, Honghong Li, Yongteng Xu, Yamei Tang

**Affiliations:** 1 Department of Neurology, Sun Yat-Sen Memorial Hospital, Sun Yat-Sen University, NO.107 Yan Jiang Xi Road, Guangzhou, Guangdong Province People’s Republic of China; 2 Department of Medical Research Center, Sun Yat-Sen Memorial Hospital, Sun Yat-Sen University, Guangzhou, Guangdong Province People’s Republic of China; 3 Guangdong Provincial Key Laboratory of Malignant Tumor Epigenetics and Gene Regulation, Sun Yat-Sen Memorial Hospital, Sun Yat-Sen University, Guangzhou, Guangdong Province People’s Republic of China; 4 Department of Radiology, Sun Yat-Sen Memorial Hospital, Sun Yat-Sen University, Guangzhou, People’s Republic of China; 5 School of Life Sciences, Sun Yat-Sen University, Guangzhou, People’s Republic of China; 6 Guangdong Province Key Laboratory of Brain Function and Disease, Zhongshan School of Medicine, Sun Yat-Sen University, Guangzhou, People’s Republic of China

**Keywords:** Brain necrosis, Radiotherapy, Radiomics model, Methylprednisolone, Treatment response, Nasopharyngeal carcinoma

## Abstract

**Background:**

Methylprednisolone is recommended as the front-line therapy for radiation-induced brain necrosis (RN) after radiotherapy for nasopharyngeal carcinoma. However, some patients fail to benefit from methylprednisolone or even progress. This study aimed to develop and validate a radiomic model to predict the response to methylprednisolone in RN.

**Methods:**

Sixty-six patients receiving methylprednisolone were enrolled. In total, 961 radiomic features were extracted from the pre-treatment magnetic resonance imagings of the brain. Least absolute shrinkage and selection operator regression was then applied to construct the radiomics signature. Combined with independent clinical predictors, a radiomics model was built with multivariate logistic regression analysis. Discrimination, calibration and clinical usefulness of the model were assessed. The model was internally validated using 10-fold cross-validation.

**Results:**

The radiomics signature consisted of 16 selected features and achieved favorable discrimination performance. The radiomics model incorporating the radiomics signature and the duration between radiotherapy and RN diagnosis, yielded an AUC of 0.966 and an optimism-corrected AUC of 0.967 via 10-fold cross-validation, which also revealed good discrimination. Calibration curves showed good agreement. Decision curve analysis confirmed the clinical utility of the model.

**Conclusions:**

The presented radiomics model can be conveniently used to facilitate individualized prediction of the response to methylprednisolone in patients with RN.

**Supplementary Information:**

The online version contains supplementary material available at 10.1186/s13014-023-02235-2.

## Introduction

In South China, nasopharyngeal carcinoma (NPC) is one of the most commonly diagnosed cancers [1]. Radiation therapy has become the mainstay of treatment for NPC, which has a long-term effect. However, the temporal lobes were inevitably exposed to radiation because the nasopharynx is located near the base of the skull. Accordingly, NPC patients treated with radiation were vulnerable to radiation-induced brain necrosis (RN), and the reported rates of RN ranged from 3 to 40% [2–6].

Currently, the treatment of RN remains challenging. Several treatment strategies have been tried for symptomatic relief, including anticoagulants, hyperbaric oxygen, vitamins, and surgery, but none of them has been shown to reverse cerebral necrosis [7–10]. The efficacy of bevacizumab has recently been suggested for cerebral radiation necrosis, but using this drug had limitation in patients with cerebral hemorrhage [11, 12]. Additionally, a previous study showed that 39.5% of patients treated with bevacizumab had a recurrence of RN [13].

For decades, intravenous steroids have been recommended as the primary therapy for RN due to their ability to reduce cytokine and inflammatory responses [14–17]. Our previous study has also shown that intravenous steroids mitigate brain necrosis in RN patients, and approximately 30% of patients had an effective response to intravenous steroids [18]. However, despite similar clinical features and therapeutic strategies, some NPC patients with RN did not benefit from steroid therapy and their brain necrosis volume might even have increased due to individual differences [15, 18]. Thus, early prediction of treatment response on steroid may further optimize clinical decision-making and improve the personalization of patient management, as well as prevent a few RN patients who might have a non-effective response from the risk of steroid-related adverse events.

Radiomics is an innovative tool which converts medical images into analytically valuable, high-dimensional features through algorithms, it uses image-based biomarkers to diagnose diseases, evaluate prognosis, and predict treatment response [19–21]. The proliferation of pattern recognition tools and the growing size of datasets have facilitated the development of radiomics, potentially improving predictive accuracy [20].

In previous studies, radiomics has been applied to predict lymph node metastasis in colorectal cancer and response to chemoradiotherapy in esophageal cancer and rectal cancer [22–24]. These studies illustrated the potential value of radiomics as a tool for predicting steroid response in brain necrosis patients. To the best of our knowledge, no radiomics-based study has been conducted for predicting the response to steroids in RN patients to date.

In the current study, we developed and validated a radiomics model for prediction of the response to intravenous methylprednisolone in patients with brain necrosis after radiotherapy for NPC.

## Methods and materials

### Patients

**Fig. 1:**
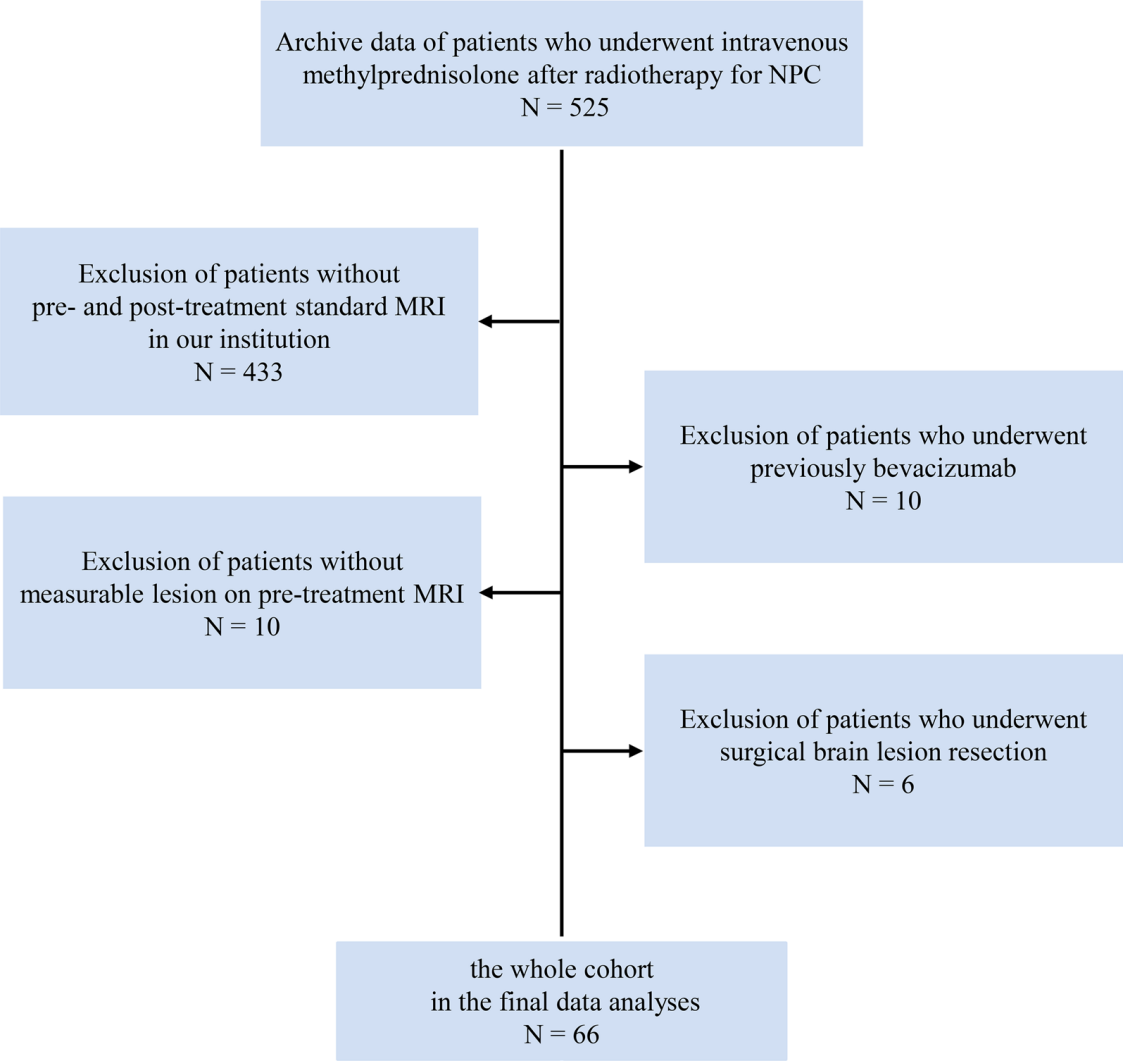
Study flowchart of cohort selection. Abbreviations: NPC = Nasopharyngeal Carcinoma; MRI = Magnetic Resonance Imaging

Ethical approval was obtained from the institutional review board for this retrospective analysis. This study comprised an evaluation of the institutional database for medical records between January 2005 and December 2016 to identify patients with brain necrosis after radiotherapy for NPC. A total of 66 NPC patients with RN were enrolled in this study, according to the following inclusion criteria: (a) underwent radiotherapy at least 12 months before the administration of intravenous methylprednisolone; (b) received high-dose or low-dose intravenous methylprednisolone treatment and no bevacizumab has been administrated before (Additional file 1: Appendix A1); and (c) performed pre- and post-treatment magnetic resonance imagings (MRI) of the brain and with measurable lesions in the MRI. The exclusion criteria were as follows: NPC relapse or metastases, surgical brain lesion resection, other tumor diseases, or other diseases of the nervous system. The patient selection process is presented in Fig. 1.

Demographic and pretreatment clinical characteristics before methylprednisolone administration were derived from medical records, including age, gender, duration between radiotherapy and RN diagnosis (DBRN), duration between radiotherapy and methylprednisolone treatment (DBRM), duration between RN diagnosis and methylprednisolone treatment (DBNM), aspartate transaminase (AST), alanine transaminase (ALT), high-sensitivity C-reaction protein levels (Hs-CRP), the maximum radiation dose of the nasopharynx (Dmax of the GTVnx), the maximum radiation dose of the neck (Dmax of the GTVnd), radiotherapy methods, Montreal Cognitive Assessment score (MoCA), the Late Effects of Normal Tissue (LENT)/Subjective, Objective, Management, Analytic (SOMA) scale score (LENT-SOMA), the volume of brain necrosis, side of the lesions and steroid dose. Tumor staging was performed on the basis of the American Joint Committee on Cancer TNM Staging System Manual, 7th Edition [25]. The RN volume was detected using T2-weighted fluid-attenuated inversion recovery (FLAIR) images 3 days before methylprednisolone administration (F0) and at 3 months (F1) of follow up. A reduction in RN volume of more than 25% at F1 compared with F0 was defined as effective response [18].

### Acquisition of MR images, segmentation of volumes of interest, and extraction of radiomic features

All patients underwent pre- and post-treatment MRI with a 1.5 T MR scanner (Gyroscan Intera; Philips, Aachen, Germany). On coronal T2-weighted FLAIR images, the brain necrosis margins can be delineated more accurately due to its high signal intensity, and the radiomics features were extracted from the images. Magnetic resonance image acquisition parameters were described in the Supplementary Information (Additional file 1: Appendix A2).

The segmentation was required before extracting quantitative radiomics features. The manual segmentation was performed using the ITK-SNAP software (Version 3.6.0; www.itk-snap.org). Two neuroradiologists blinded to the clinical data (one with 10 years of experience, another with 15 years of experience) independently delineated the regions of interest (ROIs) based on T2-weighted FLAIR images. After that, we stacked the ROIs and constructed volumes of interest (VOIs) of the brain necrosis.

Preprocessing and radiomic feature extraction were conducted using the PyRadiomics package (Version 1.3.0) in Python (3.6.4), a platform that allowed the extraction of a large panel of engineered features from images; the features and image processing can be standardised by utilizing this radiomic quantification platform [26]. A total of 961 radiomics features were extracted from T2-weighted FLAIR images using PyRadiomics. We provided the parameter settings for image processing and feature extraction to facilitate their application in Additional file 1: Appendix A3.

### Radiomics signature construction and performance assessment

We applied the least absolute shrinkage and selection operator (LASSO) method, which was suitable for regression of high dimensional data, to select treatment response-related features with nonzero coefficients [27]. Based on a linear combination of selected features, we developed a radiomics score that reflected the response to steroids for each patient. The discrimination of the radiomics signature was assessed by the area under the curve (AUC) of the receiver operator characteristic (ROC). And analyses were stratified to evaluate the performance of the radiomics signature in different subgroups according to age, gender, DBRN, and steroid dose.

### Development of an individualized prediction model

First, the radiomics signature and the clinical candidate predictors were tested in a univariate logistic regression analysis, and variables with *P* < 0.2 were subjected to subsequent multivariable analysis. The significant predictors for the prediction model were selected using a multivariate logistic regression algorithm with backward step-wise selection and Akaike’s Information Criterion (AIC) [28]. We estimated the collinearity diagnostic of multivariable logistic regression using a variance inflation factor (VIF). On the basis of the multivariate logistic regression model, a radiomics nomogram was generated.

### Performance evaluation and internal validation of predictive nomogram

A calibration curve was performed to evaluate the nomogram's calibration. The Hosmer–Lemeshow test was used to assess the goodness-of-fit of the nomogram [29]. To quantify the discrimination performance of the radiomics nomogram, AUC was measured. The radiomics nomogram was subjected to 10-fold cross-validation to calculate a relatively optimism-corrected AUC.

### Clinical use

The clinical utility of the nomogram was assessed using decision curve analysis (DCA) by calculating the net benefit to the patient based on different threshold probabilities [30].

### Statistical analysis

All statistical analyses were performed with R software (version 3.6.2; http://www.R-project.org). We used the "glmnet" package for LASSO logistic regression and the "pROC" package for plotting ROC curves. The “rms” package was used for calibration plots. The Hosmer–Lemeshow test was performed with the “generalhoslem” package. DCA plots were generated with “dca.R". Comparing the areas under the ROC curves (AUCs) of different subgroups was done with DeLong tests. All statistical tests were two-sided, and *P* values of less than 0.05 were considered significant.

## Results

### Patient clinical characteristics

As shown in Table 1, the characteristics of patients were summarized. Among all 66 RN patients, 38 patients had bilateral brain necrosis lesions (57.6%), while 28 patients (42.4%) showed unilateral brain necrosis lesions. A 3-month follow-up of T2-weighted FLAIR images showed radiological improvement in 24 (36.4%) of 66 patients, respectively. The median DBRN was 41.4 months (IQR, 32.4–57.5).

**Table 1:** Clinical characteristics of the patients by groups in the study

Variable	Patient cohort (N = 66)	Non-effective (N = 42)	Effective (N = 24)	*P* value
Age, years	49 (44–56)	49 (43–56)	50 (44–56)	0.709
Sex				0.955
Male	52 (78.8)	33 (78.6)	19 (79.2)	
Female	14 (21.2)	9 (21.4)	5 (20.8)	
Radiomics score	− 0.940 (− 1.600–0.376)	− 1.476 (− 1.927–0.935)	0.543 (0.00–1.127)	< 0.001
DBRN, months	41.4 (32.4–57.6)	41.6 (37.6–58.0)	40.0 (27.4–54.8)	0.212
DBRM, months	61.3 (43.3–76.2)	66.6 (45.4–76.2)	56.3 (41.3–77.8)	0.375
DBNM, months	7.3 (1.2–22.9)	9.0 (1.17–22.9)	6.1 (1.4–21.2)	0.689
AST, U/L	18.5 (16.0–22.3)	18.0 (16.0–21.3)	20 (16.3–25.0)	0.303
ALT, U/L	16.0 (13.0–23.0)	15.5 (14.0–23.3)	16.0 (10.0–22.8)	0.479
Hs-CRP, mg/L	2.4 (1.1–6.2)	2.4 (1.2–14.7)	2.4 (0.8–5.8)	0.594
D_max_ of the GTVnx,Gy	70.0 (70.0–72.0)	70.0 (70.0–72.0)	70.0 (70.0–73.5)	0.264
D_max_ of the GTVnd,Gy	60.0 (60.0–64.0)	60.0 (60.0–61.0)	60.0 (60.0–64.0)	0.221
Dmax of the temporal lobe, Gy	68.6 (68.6–70.6)	68.8 (68.2–70.8)	67.8 (67.2–70.0)	0.868
Radiotherapy methods				0.476
Conventional radiotherapy	58 (87.9)	36 (85.7)	22 (91.7)	
IMRT	8 (12.1)	6 (14.3)	2 (8.3)	
MOCA				0.281
< 26	47 (71.2)	28 (66.7)	19 (79.2)	
≥ 26	19 (28.8)	14 (33.3)	5 (20.8)	
LENT-SOMA^#^	5 (4–6)	5 (4–6)	5 (4–6)	0.945
NPC stage				0.905
II	6 (9.1)	5 (11.9)	1 (4.2)	
III	26 (39.4)	14 (33.3)	12 (50.0)	
IV	21 (31.8)	15 (35.7)	6 (25.0)	
IVA	13 (19.7)	8 (19.1)	5 (20.8)	
RN volume at baseline, cm^3^	26.4 (8.8–59.5)	26.4 (8.3–64.2)	25.7 (8.9–45.2)	0.759
Side of FLAIR lesions				0.259
Unilateral	28 (42.4)	20 (47.6)	8 (33.3)	
Bilateral	38 (57.6)	22 (52.4)	16 (66.7)	
Steroid dose, mg/d				0.244
80	31 (47.0)	22 (52.4)	9 (37.5)	
500	35 (53.0)	20 (47.6)	15 (62.5)	

Data are shown as numbers (%) or medians (interquartile ranges). *P* value is calculated from chi-square test for categorized variables and two-sample t-test/Mann–Whitney U test for continues variables, which represents the univariate association test of subgroups

*DBRN*, duration between radiotherapy and radiation-induced brain necrosis (RN) diagnosis; *DBRM*, duration between radiotherapy and methylprednisolone treatment; *DBNM*, duration between RN diagnosis and methylprednisolone treatment; *AST*, aspartate transaminase; *ALT*, alanine transaminase; *Hs-CRP*, high-sensitivity C-reaction protein levels; *Dmax of the GTVnx*, the maximum radiation dose of the nasopharynx; *Dmax of the GTVnd*, the maximum radiation dose of the neck; *IMRT*, intensity-modulated radiation therapy; *MoCA*, Montreal Cognitive Assessment score; *LENT-SOMA*, the late effects of normal tissue (LENT)/subjective, objective, management, analytic (SOMA) scale score; *NPC*, nasopharyngeal carcinoma; *RN*, radiation-induced brain necrosis; *FLAIR*, fluid-attenuated inversion recovery

^#^ summary grade of the Subjective, Objective, and Management(SOM) characteristics

### Radiomics signature construction and performance assessment

**Fig. 2:**
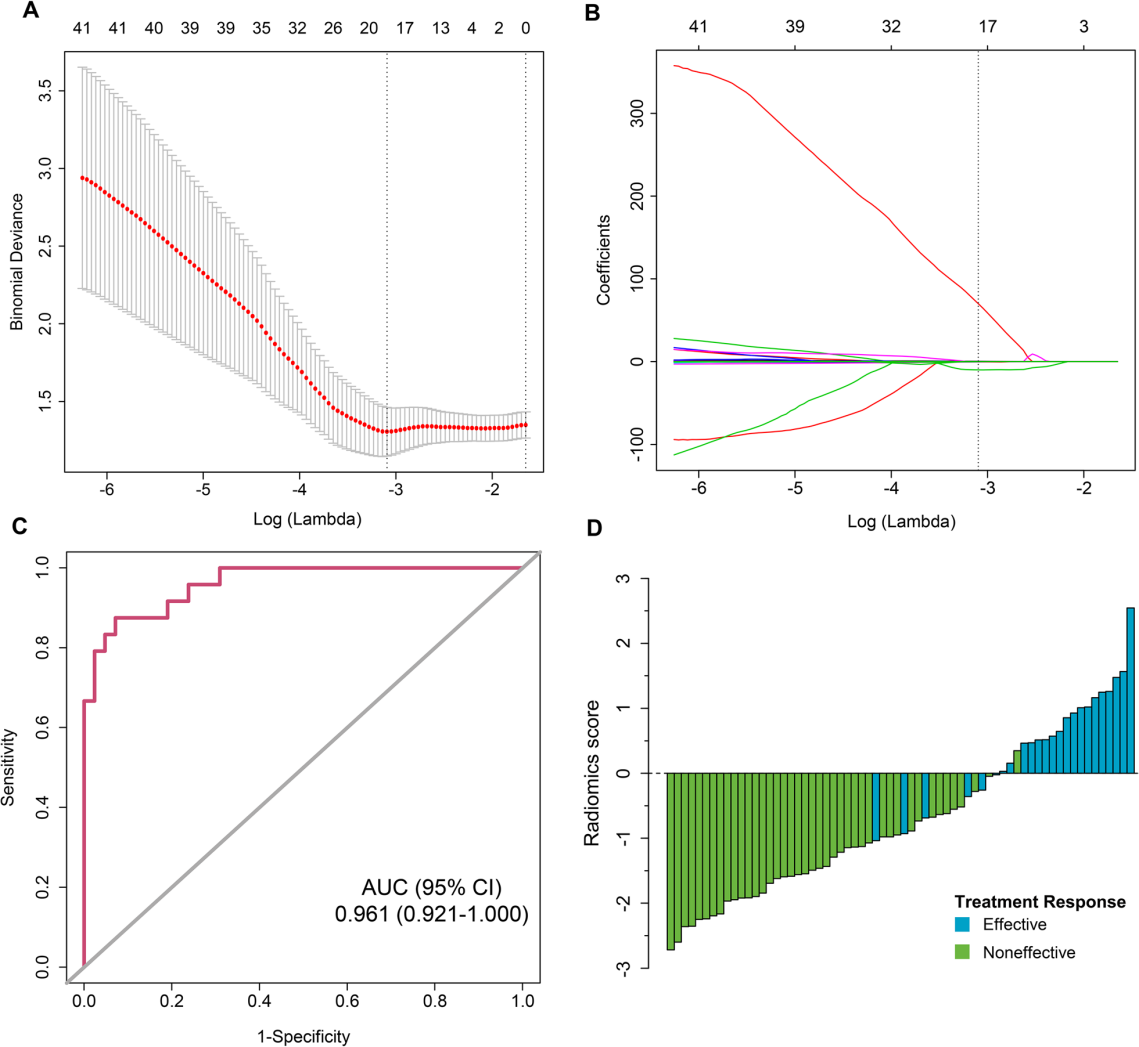
Radiomics feature selection using LASSO binary logistic regression and the performance of the radiomics signature. **A** Tuning parameter (λ) selection in the LASSO model used tenfold cross-validation via minimum criteria. The binomial deviance was plotted versus log (λ). The dotted vertical lines were drawn at the optimal λ values based on the minimum criteria and 1 standard error of the minimum criteria. The optimal λ value of 0.0454 with log (λ) =  − 3.093 was selected. **B** LASSO coefficient profiles of the 961 radiomics features. The dotted vertical line was drawn at the λ value of 0.0454, where optimal λ resulted in 16 nonzero coefficients. Plots **C** present the ROC curves of the radiomics signature in the 66 patients, respectively. **D** Waterfall plot for distribution of radiomics score and response to steroid of individual patients. Green bars show scores for patients who experienced progression, while blue bars show scores for those who exhibited radiological improvement

We extracted a total of 961 radiomics features of necrosis lesions based on T2-weighted FLAIR images. According to LASSO logistic regression, 16 features with nonzero coefficients were screened (Fig. 2A and B). These features were incorporated into the radiomics score calculation formula (Additional file 1: Appendix A4).

The radiomics signature achieved good discrimination for predicting the response to steroids in RN patients, with an AUC of 0.961 (95% CI, 0.921–1.000, Fig. 2C). The waterfall plot revealed the distribution of radiomics scores and treatment responses for individual patients (Fig. 2D). The radiomics signature achieved good discrimination in the stratified analysis according to age, gender, DBRN, and steroid dose. And the DeLong test results of AUCs showed no significant difference in the different subgroups (Additional file 2: Fig. S1).

### Development of an individualized prediction model

**Fig. 3:**
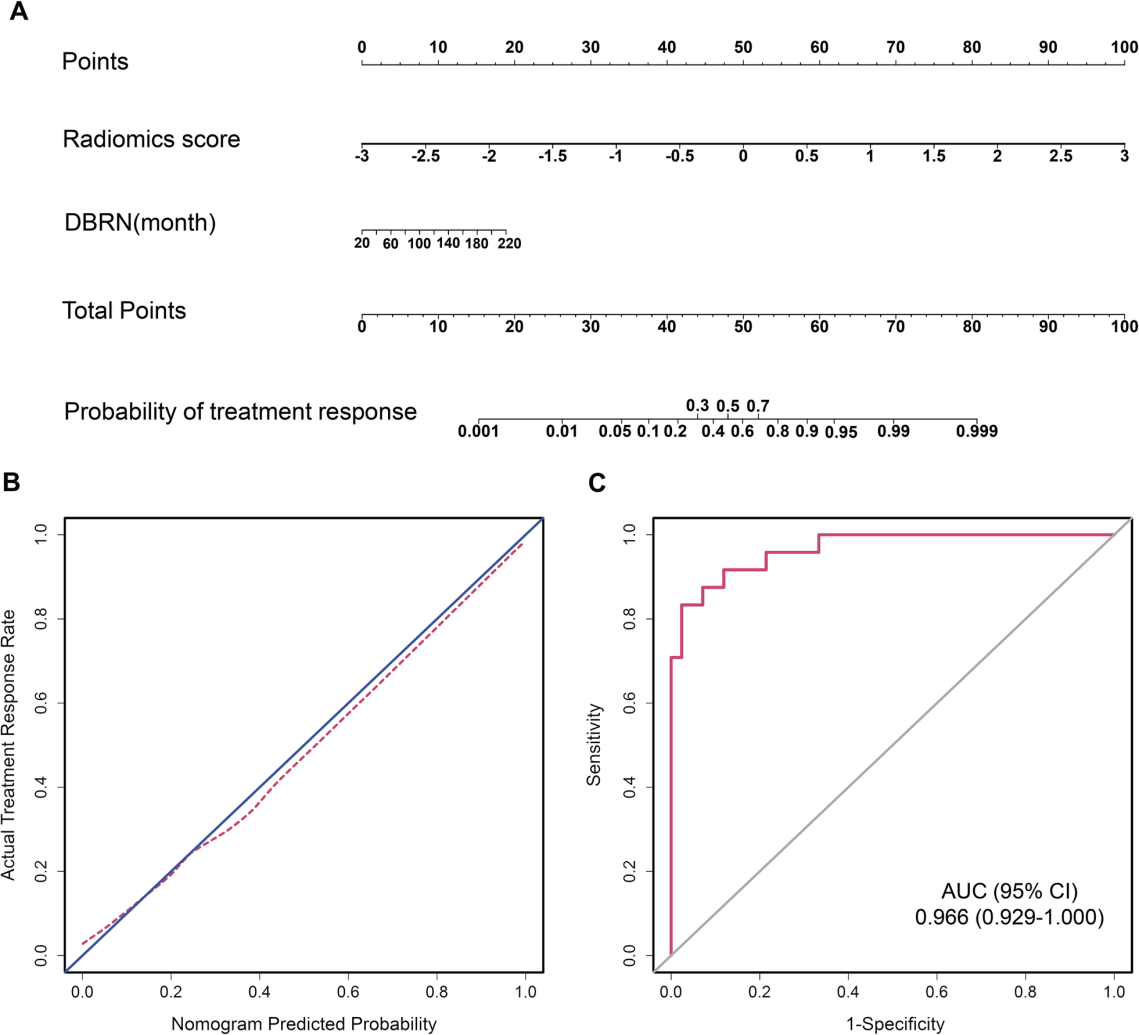
Radiomics nomogram for the prediction of therapeutic response to steroid and the performance of the nomogram. **A** Radiomics nomogram based on radiomic signatures and clinical factor. Plots **B** shows the calibration curve of the nomogram. Plot **C** presents the ROC curve of the radiomics nomogram

Univariate logistic regression analysis revealed that four variables, consisting of the radiomics signature, MoCA scores, LENT-SOMA scores, and DBRN, were significant at a level of *P* < 0.2 (Table 2). A multivariate logistic regression analysis identified the radiomics signature and DBRN as independent predictors (Table 2). The radiomics score (per 0.1 increase) remained a strong independent predictor of response to steroids after adjusting for clinical factors (OR 3.388, 95% CI, 0.538–21.334, *P* < 0.001). With regard to the collinearity diagnosis, the VIF values of the four predictive factors ranged from 1.022 to 1.065, which indicated no collinearity. A model that incorporated the radiomics signature and DBRN was developed and presented as the nomogram (Fig. 3A).

**Table 2:** Potential predictors of the response to steroid in patients with brain necrosis

Variable	Univariate logistic regression		Multivariate logistic regression	
	OR (95%CI)	P*	OR (95%CI)	*P*
Age, years	1.008 (0.954–1.065)	0.774		
Sex		0.955		
Male	Reference			
Female	0.965 (0.282–3.302)			
Radiomics score (per 0.1 increase)	2.172 (0.476–9.916)	< 0.001^*^	3.388 (0.538–21.334)	< 0.001
DBRN, months	0.992 (0.983–1.000)	0.050^*^	1.020 (1.001–1.040)	0.034
DBRM, months	0.997 (0.981–1.012)	0.664		
DBNM, months	0.992 (0.958–1.028)	0.674		
AST, U/L	1.029 (0.939–1.128)	0.543		
ALT, U/L	0.989 (0.942–1.038)	0.655		
Hs-CRP, mg/L	0.999 (0.978–1.021)	0.947		
D_max_ of the GTVnx,Gy	1.130 (0.910–1.404)	0.269		
D_max_ of the GTVnd,Gy	1.082 (0.947–1.237)	0.246		
Dmax of the temporal lobe, Gy	0.982 (0.827–1.167)	0.838		
Radiotherapy methods		0.481		
Conventional radiotherapy	Reference			
IMRT	0.545 (0.101–2.944)			
MOCA		0.048^*^		
< 26	Reference			
≥ 26	0.357 (0.129–0.992)			
LENT-SOMA^#^	0.917 (0.840–1.000)	0.050^*^		
NPC stage		0.469		
II	Reference			
III	4.286 (0.438–41.954)	0.211		
IV	2.000 (0.191–20.898)	0.563		
IVA	3.125 (0.278–35.157)	0.356		
Steroid dose, mg/d		0.246		
80	Reference			
500	1.833 (0.658–5.107)			

*DBRN*, duration between radiotherapy and radiation-induced brain necrosis (RN) diagnosis; *DBRM*, duration between radiotherapy and methylprednisolone treatment; *DBNM*, duration between RN diagnosis and methylprednisolone treatment; *AST*, aspartate transaminase; *ALT*, alanine transaminase; *Hs-CRP*, high-sensitivity C-reaction protein levels; *Dmax of the GTVnx*, the maximum radiation dose of the nasopharynx; *Dmax of the GTVnd*, the maximum radiation dose of the neck; *IMRT*, intensity-modulated radiation therapy; *MoCA*, montreal cognitive assessment score; *LENT-SOMA*, the late effects of normal tissue (LENT)/subjective, objective, management, analytic (SOMA) scale score; *NPC*, nasopharyngeal carcinoma

*P < 0.02; ^#^summary grade of the Subjective, Objective, and Management (SOM) characteristics

### Performance evaluation and internal validation of predictive nomogram

The calibration curve of the radiomics nomogram for the probability of benefit from steroid treatment showed favorable agreement between prediction and observation in the study (Fig. 3B). The Hosmer–Lemeshow test indicated good calibration power, with a non-significant *P* value of 0.818. The radiomics nomogram revealed good discrimination, with an AUC of 0.966 (95% CI, 0.929–1.000, Fig. 3C) and an optimism-corrected AUC of 0.967 via 10-fold cross-validation.

### Clinical use

**Fig. 4:**
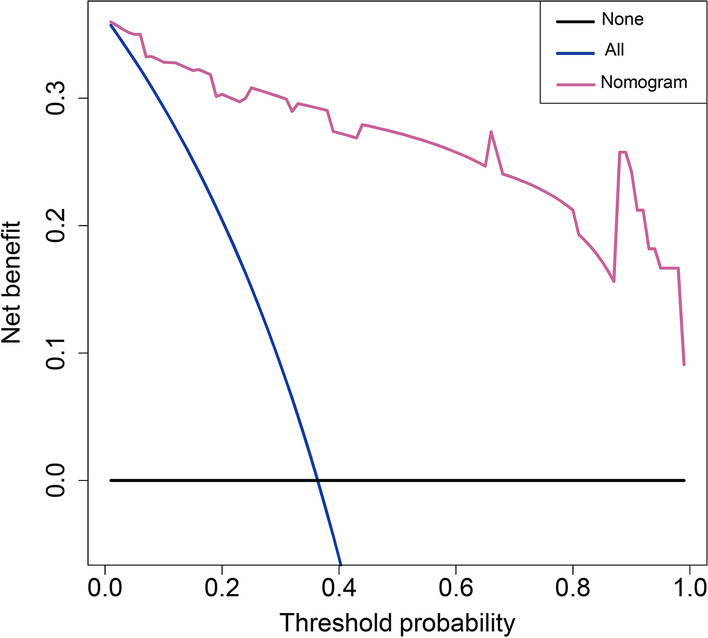
DCA for the radiomics nomogram. The Y-axis represents the net benefit. The X-axis represents the threshold probability. The threshold probability is where the expected benefit of treatment is equal to the expected benefit of avoiding treatment. The pink line represents the radiomics nomogram. The blue line represents the hypothesis that all patients had benefit from steroid treatment. The black line represents the hypothesis that no patients had benefit from steroid treatment. For example, if the possibility of benefit from steroid treatment in a patient is over the threshold probability, then a treatment strategy for steroid treatment benefit should be adopted. The decision curves demonstrate that if the threshold probability was between 0 and 1, then using the radiomics nomogram to predict benefit from steroid treatment added more benefit than treating either all or no patients. Abbreviations: DCA, decision curve analysis

Figure 4 illustrated the results of the decision curve analysis for the radiomics model. With regard to clinical application, the DCA demonstrated favorable performance for the model. While the probability of achieving effective response ranged from 0 to 100%, using the radiomics nomogram to determine effective response to steroid showed a greater advantage than either the regimen in which all patients were assumed to achieve effective response or the regimen in which no patients were.

## Discussion

In the current study, we developed and validated a radiomics signature-based nomogram that incorporating the radiomics signature and the clinical variable for individualized prediction of the response to steroids in NPC patients with RN. This study demonstrated that radiomics features from MRI images can be used to predict early therapeutic effects of steroids in RN patients and provided a non-invasive pre-treatment prediction tool to identify RN patients with a high probability of therapeutic benefit to steroids.

With the mechanisms underlying brain necrosis after radiotherapy being revealed gradually, the direct injury to endothelial and glial cells has been given priority in the study of its relationship with brain necrosis, which resulted in demyelination and vascular hyalinization. This primary pathology caused tissue inflammation and ischemia, resulting in numerous tissue protective responses such as angiogenesis [31]. Previous studies have indicated that corticosteroids could suppress cytokine and inflammatory responses, reducing brain necrosis and changes in blood vessels and inflammation [14, 17]. Therefore, steroids have been recommended as the primary therapy in RN patients for decades [14].

However, steroid treatment may not be beneficial for all patients, and it is currently difficult to determine which patients will benefit from it. Furthermore, some patients might suffer from steroid-related adverse events such as infections, hyperglycemia, osteoporosis, peptic ulcer disease and liver damage [32, 33]. Thus, it is essential to develop an accurate predictive tool for the pre-treatment prediction of therapeutic effects to steroids in RN patients. And if the patients who are at high probability of therapeutic benefit to steroids can be identified before treatment, then these patients might be good candidates for intravenous steroids treatment.

Recent advances in radiomics have led to new insights into personalized medical care in cancer practices that taked into account tumor diagnosis, classification of subtypes, and treatment response prediction [21, 24, 34]. Findings from these studies emphasized the significance of radiomics, which can also be used to identify patients with a high probability of benefitting from steroid therapy.

Consequently, we aimed to select the key radiomics features from MRI images and construct a radiomics signature in this study. Our radiomics signature exhibited favorable discrimination across the whole data set. Encouragingly, the radiomics signature also demonstrated favorable discrimination in stratified analysis according to age, gender, DBRN, and steroid dose.

Then, we identified the radiomics signature and DBRN as independent significant predictors based on a multivariate logistic regression model, with corresponding odds ratios of 3.3876 and 1.020. The odds ratio for DBRN suggested that the later brain necrosis occurred after radiotherapy, the higher the probability of steroid-related therapeutic benefit. This finding might largely be attributed to the mechanism of steroid on radiation-induced brain necrosis. Prior studies have demonstrated that early brain necrosis after irradiation was generally attributed to transient demyelinating processes related to blood–brain barrier injury or selective oligodendrocyte dysfunction [35, 36]. Thus, steroids failed to alleviate edema at early stage significantly. While during the late brain necrosis phase, radiation of the brain resulted in multiple inflammatory changes. Numerous inflammatory cells, such as macro-phages and lymphocytes, were detected with telangiectatic vascularization in the area surrounding the necrosis and might serve as potential targets for successful corticosteroid therapy [16, 37]. Hence, the patients who occurred brain necrosis later after radiotherapy might have a greater probability of favorable response to steroids.

In order to provide clinicians with an easy-to-use tool, we constructed a radiomics nomogram that integrating the radiomics signature and DBRN for prediction of the response to steroids, which showed satisfactory discrimination with an AUC of 0.966. DCA was generally used to evaluate whether the radiomics model-based decisions could benefit patients based on threshold probability [28, 38]. In our study, DCA revealed that if the threshold probability varied from 0 to 100%, applying the radiomics nomogram to determine effective response to steroid showed a higher overall net benefit than either the treat-all or the treat-none scheme. Therefore, the patients who were designated to benefit from steroid applying our radiomics nomogram had a comparatively high possibility of receiving true therapeutic benefit from steroids, and intravenous methylprednisolone would be recommended in these patients, especially for the patients who occurred brain necrosis later after radiotherapy. In addition, surgical operation and bevacizumab were reserved for steroid refractory brain necrosis.

This is the first attempt to develop a radiomics signature-based nomogram for predicting therapeutic effects of steroids in NPC patients with RN. Overall, our study has two strengths. First, high-dimensional features extracted from MRI in the current study provide more detailed information about brain lesions, which can improve the accuracy and robustness of prognostic model. Furthermore, the radiomics feature extraction was conducted on three-dimensional volumes of interest (VOIs) of brain lesions rather than the two-dimensional regions of interest (ROIs) of brain lesions, which can better reflect the heterogeneity of the entire lesion. Second, the presented radiomic model is composed of only two items, they are available from routine MRI analysis and clinical data. Therefore, our prognostic model may serve as a non-invasive tool for the pre-treatment prediction of the response to steroids in NPC patients with RN.

Despite its strengths, some limitations still exist. First, our study is the lack of validation in an external cohort. A larger sample size from multiple centers should be investigated to validate the robustness and reproducibility of our proposed radiomics. Second, given the retrospective nature of this study, long-term follow-up data is not available for assessing whether the patients benefitting from steroids exhibit recurrence, which could help clinical decision-making. Thus, further studies are necessary to resolve these issues.

In summary, our study presents a radiomics nomogram incorporating both the radiomics signature and clinical variable, which can be conveniently used to predict therapeutic effects to intravenous steroids in NPC patients with RN. Further external validation is required to evaluate the predictive ability of the radiomics model prior to its implementation in clinical application.

## Supplementary Information

**Additional file 1** Supplementary material: Appendices.

**Additional file 2** Supplementary figures.

## Data Availability

The datasets generated during and analysed during the current study are available from the corresponding author on reasonable request.

## Abbreviations

RN: Radiation-induced brain necrosis
NPC: Nasopharyngeal carcinoma
LASSO: Least absolute shrinkage and selection operator
MRI: Magnetic resonance imaging
DBRN: Duration between radiotherapy and radiation-induced brain necrosis diagnosis
DBRM: Duration between radiotherapy and methylprednisolone treatment
DBNM: Duration between radiation-induced brain necrosis diagnosis and methylprednisolone treatment
AST: Aspartate transaminase
ALT: Alanine transaminase
Hs-CRP: High-sensitivity C-reaction protein levels
Dmax: Maximum dose
GTVnx: Radiation dose of the nasopharynx
GTVnd: Radiation dose of the neck
MoCA: Montreal cognitive assessment score
LENT-SOMA: Late effects of normal tissue/subjective, objective, management, analytic scale score
FLAIR: Fluid-attenuated inversion recovery
ROIs: Regions of interest
VOIs: Volumes of interest
AUC: Area under the curve
ROC: Receiver operator characteristic
AIC: Akaike’s information criterion
VIF: Variance inflation factor
DCA: Decision curve analysis
IMRT: Intensity-modulated radiation therapy
