# Supplementary material for: A radiomics model for predicting the response to methylprednisolone in brain necrosis after radiotherapy for nasopharyngeal carcinoma
Source: Radiat Oncol. 2023 Mar 1;18:43. doi: 10.1186/s13014-023-02235-2 (PMC9979431; doi:10.1186/s13014-023-02235-2)
Supplement: Supplementary file 1 — Additional file 1 Supplementary material: Appendices. [file 13014_2023_2235_MOESM1_ESM.docx]

**Supplementary materials**

**Appendix A1: Methylprednisolone regimens**

Before 2009, patients with brain necrosis after radiotherapy for NPC were treated with low-dose methylprednisolone ^[1, 2]^. Low-dose methylprednisolone was administered as an intravenous infusion of 1 mg/kg/day for 5 consecutive days, then 40 mg for 5 days, then oral prednisone 30 mg per day, gradually tapering by 5 mg/week to a maintenance dose of 10 mg daily for 3 months. All patients with brain necrosis after radiotherapy for NPC were generally treated with high methylprednisolone after 2009^[3]^. High-dose methylprednisolone was administered as an intravenous infusion of 500 mg for 3 consecutive days, 80 mg for 4 days, 40 mg for 4 days, then oral prednisone 30 mg/day, gradually tapering by 5 mg/week to a maintenance dose of 10 mg daily for 3 months.

**Appendix A2: The Information of the MRI acquisition parameters**

The T2-weighted FLAIR images acquisition parameters were as follows: slice thickness, 5 mm; gap, 1.5 mm; echo time, 180 ms; repetition time, 11,000 ms; inversion time, 2,800 ms; and echo train length, 48.

**Appendix A3: Radiomic Feature Extraction**

The analysis presented in the manuscript used features extracted using PyRadiomics

version v1.3.0. The feature definitions below reflect this version of PyRadiomics. The

current version of PyRadiomics is available online. For code see

<https://github.com/Moo-YewTsing/Edema-Prediction>

**Appendix A4: Radiomics score (Rad-score) calculation formula:**

Rad score =

-6.04119309

-original_firstorder_Kurtosis * 0.42430453

+original_firstorder_Skewness * 0.31665209

+original_glcm_MaximumProbability * 69.50099028

-wavelet.HHH_firstorder_Kurtosis * 0.04497318

+wavelet.HHH_firstorder_Median * 0.23803082

-wavelet.HHH_glcm_Idn * 10.00314415

-wavelet.HHL_firstorder_Skewness * 0.17460345

-wavelet.HLH_glcm_ClusterShade * 0.01506396

-wavelet.HLL_firstorder_90Percentile * 0.00819933

-wavelet.HLL_glszm_SmallAreaHighGrayLevelEmphasis * 0.00018852

+wavelet.LHH_firstorder_Mean * 0.11501556

+wavelet.LHH_firstorder_Median * 0.07371388

-wavelet.LHH_glcm_Correlation * 0.10810974

+wavelet.LHL_firstorder_Maximum * 0.01295037

+wavelet.LHL_firstorder_RootMeanSquared * 0.04163792

+wavelet.LHL_glszm_LargeAreaHighGrayLevelEmphasis * 0.00000510

**Appendix Figures:**

**Figure S1. Stratified analysis of the radiomics signature**

Radiomic signature for each subgroup stratified by (A) age, (B) steroid dose, (C) gender, and (D) DBRN. The DeLong test was performed to compare the areas under the ROC curves (AUCs) in different subgroups.

The results showed that our radiomic signature was not influenced by these factors (all *p*>0.05), indicating a good generalizability of the radiomic signature (see Figure S1).

[1] Goodkin D E, Kinkel R P, Weinstock-Guttman B, et al. A phase II study of i.v. methylprednisolone in secondary-progressive multiple sclerosis[J]. Neurology. 1998;51:239-245.

[2] Beck R W, Cleary P A, Trobe J D, et al. The effect of corticosteroids for acute optic neuritis on the subsequent development of multiple sclerosis. The Optic Neuritis Study Group[J]. N Engl J Med. 1993;329:1764-1769.

[3] Tang Y, Rong X, Hu W, et al. Effect of edaravone on radiation-induced brain necrosis in patients with nasopharyngeal carcinoma after radiotherapy: a randomized controlled trial[J]. J Neurooncol. 2014;120:441-447.
