# Supplementary figures and images for: A radiomics model for predicting the response to methylprednisolone in brain necrosis after radiotherapy for nasopharyngeal carcinoma
Source: Radiat Oncol. 2023 Mar 1;18:43. doi: 10.1186/s13014-023-02235-2 (PMC9979431; doi:10.1186/s13014-023-02235-2)

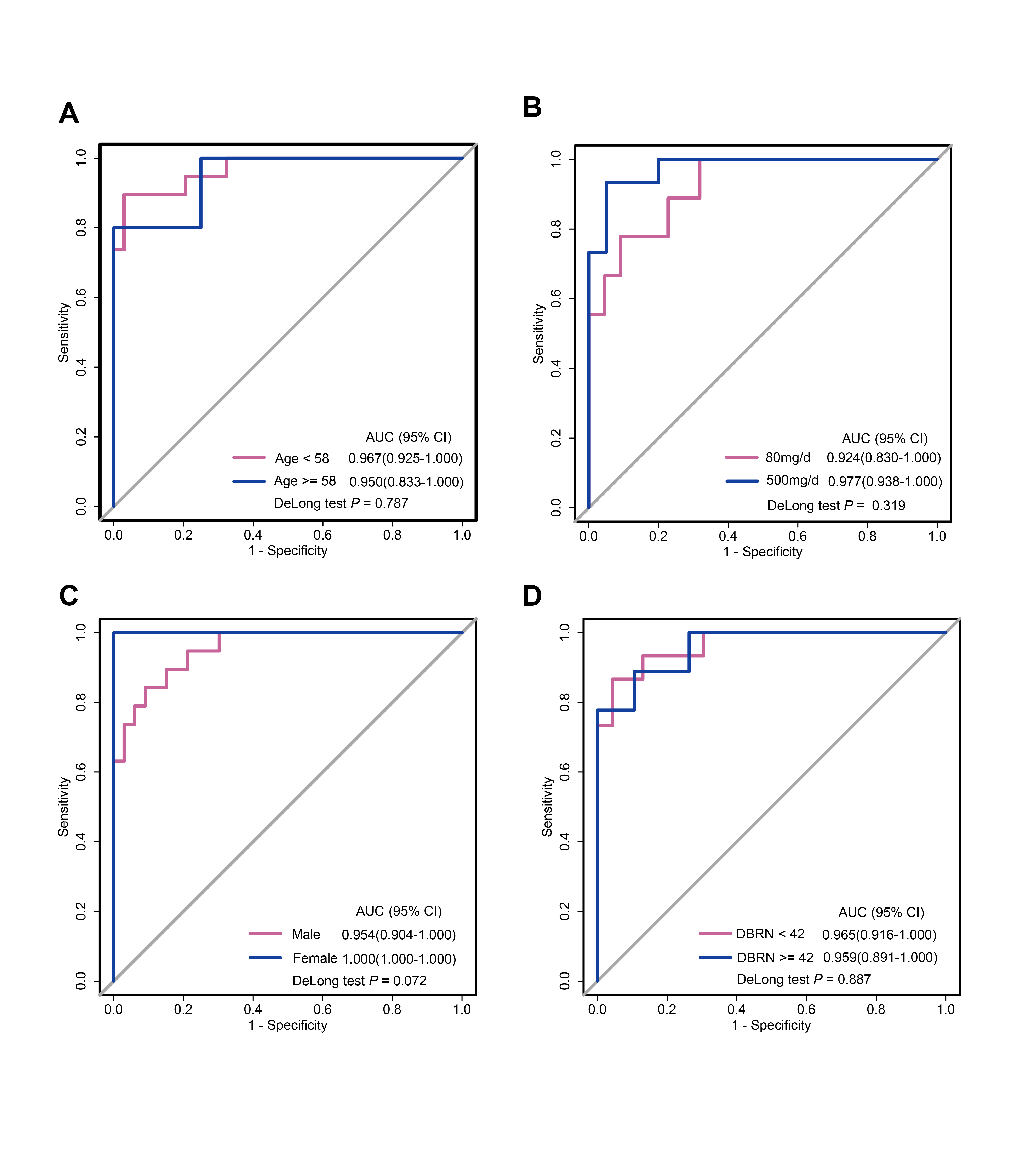

Supplement: Supplementary file 2 — Additional file 2 Supplementary figures. [file 13014_2023_2235_MOESM2_ESM.tif]
